# Editorial: Spatial Navigation and Neurodevelopmental Disorders

**DOI:** 10.3389/fpsyt.2022.875868

**Published:** 2022-03-24

**Authors:** Noemi Faedda, Laura Piccardi, Maddalena Boccia, Åsa Bartonek, Cecilia Guariglia

**Affiliations:** ^1^Department of Human Neuroscience, Section of Child and Adolescents Neuropsychiatry, Sapienza University of Rome, Rome, Italy; ^2^Department of Psychology, University Sapienza of Rome, Rome, Italy; ^3^Cognitive and Motor Rehabilitation and Neuroimaging Unit, Istituto di Ricovero e Cura a Carattere Scientifico (IRCCS) Fondazione Santa Lucia, Rome, Italy; ^4^Neuropaediatric Unit, Department of Women's and Children's Health, Karolinska Institutet, Stockholm, Sweden

**Keywords:** spatial navigation, executive function, neurodevelopmental disorders, attention deficit and hyperactivity disorder (ADHD), down syndrome

Spatial navigation is one of the most complex cognitive skills. For a long time, several authors have tried to delineate its development during the life span, finding as navigational abilities develops at different times (1, 2). By the age of 6–9 months, children are able to move in the environment using only egocentric strategies [e.g., (3, 4)]. At 11 months they began to use landmarks information and landmark arrays and by the end of the first year of life children are able to track their own movements to locate formerly visited places in the environment after moving to a new location (5). Between 18 and 24 months of age they can find hidden objects using geometric environmental information (6, 7) or a combination of visual landmarks and information derived from their own movements (8); at this age, they are also able to re-orient themselves according to the geometric shape of a room, but are unable to integrate this information with other clearly perceivable visual cues (9, 10). Spatial navigation requires a wide repertoire of cognitive abilities, such as attention, memory, perception, and decision-making, and it is well known that impairment of one or more of these abilities negatively affects this process (11). Thus, a slight delay in the development of one of the cognitive functions involved in spatial navigation may result in a navigational impairment such as to get lost in both familiar and unfamiliar places, or to be unable to perceive and identify familiar buildings and landscapes. This Research Topic aimed to understand how the development of spatial navigation skills in children and adolescents is dependent on the development of other cognitive domains, in particular executive functioning. The term executive functioning refers to a series of higher cognitive skills, fundamental for the proper functioning of daily activities. A deficit in executive functioning can lead to poor attention, planning difficulties, difficulty in generating and implementing navigational strategies. To this purpose all studies collected in this Research Topic have addressed different aspects of spatial navigation in different clinical populations as well as in the typical development (TD).

Four studies included clinical populations compared with children with TD, while one study describes a single case affected by developmental topographical disorientation (DTD) and another study focused on aspects of navigational abilities in Down and William Syndromes by reviewing literature.

In detail, Faedda et al. evaluated the topographic memory in children with ADHD-combined subtype (ADHD-C) showing that in ADHD-C children initial topographic learning was compromised differently from the long-term retention of learned topographical material. Specifically, this deficit seemed to be linked with difficulties in sustained attention, in spatial memory for novel visual materials and in working memory, but also due to perseverative behaviors.

Bartonek et al. explored topographic working memory in children with myelomeningocele (MMC) and arthrogryposis multiplex congenital (AMC) compared to a group of children with TD finding that the topographic working memory span was shorter in the MMC group than in the TD group as well as shorter in both MMC and AMC groups than TD when analyzed according to their functionally used ambulation.

Meneghetti et al. analyzed path learning and the conditions favoring it, as well as the cognitive abilities involved in navigational skills of individuals with Down syndrome (DS). Their results showed that path learning in DS group depends on experimental conditions. They performed well when they observe the experimenter's moves, differently their performance is affected by verbal instructions.

Castilla et al. designed and tested a new protocol named the “Virtual House Locomotor Maze” (VHLM) for studying inhibitory control as well as mental flexibility using a visuo-spatial locomotor memory test. The authors examined planning and replanning abilities to take the shortest path to reach a target house in a sample of TD children. Their results suggest that several strategies are used for replanning and these measures could be used as an index of impulsivity.

Del Lucchese et al. analyzed a new ecological navigation task, the Virtual City paradigm™ (VC™) to test visuo-spatial memory and executive functions in children with ADHD. The VC™ consisting of a virtual town with houses, streets and crossroads projected on the ground. In one condition, children were required to walk through the city and reach a sequence of houses. In the other, before walking, they had to plan the shortest path to reach the houses, inhibiting the prepotent response to start walking. The results show a good feasibility of the paradigm (feasibility checklist and ad hoc questionnaire), being ecological and motivating. VC™ measures of span positively correlated with visuo-spatial short and working memory measures, suggesting that VC™ heavily relies on efficient spatial memory.

Rusconi et al. reported a 22-year-old woman with a normal development and no clinical history of neurological or psychiatric diseases suffering from DTD. She has been evaluated twice, with an interval of 5 years. The MRI did not reveal any morphological alteration, while diffusion tensor imaging (DTI) showed a structural connectivity deficit in the parieto–prefrontal and parieto–premotor pathway. She suffered from different deficits in spatial and navigational tasks. Moreover, she showed a poor performance in high-level face encoding and retrieval sustaining that DTD can co-occur with deficits in face recognition.

Lavenex and Lavenex performed a review on spatial abilities in Down and Williams Syndromes (WS) discussing their recent works. They found that DS may use egocentric route learning without integrating individual landmarks and a low-resolution allocentric spatial representation encoding the relationships between different locations. In contrast, most individuals with WS are unable to build or use a low-resolution allocentric or configural representation of the environment. However, they may use visually and verbally encoded landmarks as beacons to learn routes. They also discussed the main neural structures involved in these different spatial processes and explain how the relative preservation or impairment of specific brain functions may engender the unique cognitive profiles observed in individuals with DS and WS.

## Author Contributions

All authors listed have made a substantial, direct, and intellectual contribution to the work and approved it for publication.

## Conflict of Interest

The authors declare that the research was conducted in the absence of any commercial or financial relationships that could be construed as a potential conflict of interest.

## Publisher's Note

All claims expressed in this article are solely those of the authors and do not necessarily represent those of their affiliated organizations, or those of the publisher, the editors and the reviewers. Any product that may be evaluated in this article, or claim that may be made by its manufacturer, is not guaranteed or endorsed by the publisher.
